# Antimicrobial and antioxidant properties of methanol extract, fractions and compounds from the stem bark of *Entada abyssinica *Stend ex A. Satabie

**DOI:** 10.1186/1472-6882-11-57

**Published:** 2011-07-19

**Authors:** Gerald N Teke, Paul K Lunga, Hippolyte K Wabo, Jules-Roger Kuiate, Gerard Vilarem, Geraldine Giacinti, Haruhisa Kikuchi, Yoshiteru Oshima

**Affiliations:** 1 Laboratory of Microbiology and Antimicrobial Substances, Faculty of Science, University of Dschang, P.O. Box 67 Dschang, Cameroon; 2 Department of Chemistry, Faculty of Science, University of Dschang, P.O. Box 67, Dschang, Cameroon; 3 Laboratoire de Chimie Agro-Industrielle - UMR 1010 INRA/INP-ENSIACET 4, Allée Emile Monso 31432 Toulouse Cedex 4, France; 4 Graduate School of Pharmaceutical Sciences, Tohoku University, 6-3 Aoba, Aramaki, Aoba-ku, Sendai 980-8578, Japan

## Abstract

**Background:**

The aim of this study was to evaluate the antimicrobial and antioxidant activities of the methanol extract, fractions and isolated compounds from *Entada abyssinica *stem bark, plant used traditionally against gastrointestinal infections.

**Methods:**

The methanol extract of *E. abyssinica *stem bark was pre-dissolved in a mixture of methanol and water, and then partitioned between *n*-hexane, ethyl acetate and *n*-butanol. The ethyl acetate portion was fractionated by column chromatography and the structures of isolated compounds elucidated by analysis of spectroscopic data and comparison with literature data. Antimicrobial activity was assayed by broth microdilution techniques on bacteria and yeasts. The antioxidant activity was determined by DPPH radical scavenging method.

**Results:**

Four known compounds [(5*S*,6*R*,8a*R*)-5-(carboxymethyl)-3,4,4a,5,6,7,8,8a-octahydro-5,6,8a-trimethylnaphthalenecarboxylic acid (**1**), methyl 3,4,5-trihydroxybenzoate (**2**), benzene-1,2,3-triol (**3**) and 2,3-dihydroxypropyltriacontanoate (**4**)] were isolated. Compared to the methanol extract, fractionation increased the antibacterial activities of the *n*-hexane and ethyl acetate fractions, while the antifungal activities increased in ethyl acetate, *n*-butanol and aqueous residue fractions. The isolated compounds were generally more active on bacteria (9.7 to 156.2 μg/ml) than yeasts (78.1 to 312.5 μg/ml). Apart from compound **1**, the three others displayed DPPH^· ^scavenging activity (RSa), with RSa_50 _values of 1.45 and 1.60 μg/ml.

**Conclusion:**

The results obtained from this study support the ethnomedicinal use of *E. abyssinica *in the treatment of gastrointestinal infections and the isolated compounds could be useful in the standardisation of antimicrobial phytomedicine from this plant.

## Background

Exploring the healing power of plants is an ancient concept. For many centuries people have been trying to alleviate and treat diseases with different plant extracts and formulations [1]. The interest in plants with antimicrobial properties has been revived because of current problems associated with the use of antibiotics [2]. Many microbial infections lead to the production of highly reactive molecules from the metabolism of oxygen that can cause extensive damage to cells and tissues [3]. The fact that microorganisms nowadays tend to develop resistance towards drugs, coupled to the undesirable side effects of certain antibiotics offer considerable potentials for the development of new effective antimicrobial and antioxidant agents; medicinal plants are a prolific source.

*Entada abyssinica *(Mimosaceae) is an understorey forest deciduous tree, 3-15 m high, with a flat spreading crown. It is widespread in central and eastern tropical Africa [4]. It has a grey stem bark, glabrous leaves and creamy white flowers [5]. A decoction of the stem bark is locally used in the treatment of coughs, rheumatic and abdominal pains, and diarrhoea while the root or leaf decoction is used in the treatment of fever and to prevent miscarriage [5]. The fresh roots are used against gonorrhoea [6].

Some biological activities of *Entada abyssinica *have been reported. The leaves are highly active against Semliki forest virus [7]. Weak antibacterial activities of the methanol extract of *E. abyssinica *stem bark has been reported by Fabry et al. [8]. A number of biologically active compounds have been isolated from *E. abyssinica *including a diastereoisomer of the clerodane type diterpene, kolavenol [9], flavonoids and phytosterol glycosides [10] and kolavic acid derivatives [11].

However, to the best of our knowledge, no information on its free radical scavenging activities is available. This study was therefore designed to evaluate the possible beneficial antimicrobial and antioxidant potencies of the methanol extract, fractions and compounds from this plant.

## Methods

### Plant material, extraction and phytochemical screening

The stem bark of *E. abyssinica *was collected in May 2007 in Menoua Division, West Cameroon. Botanical identification was done at the Cameroon National Herbarium in Yaounde by Mr Tadjouteu Fulbert, where a voucher specimen was kept under the reference number 44732/HNC. The stem bark was cut into pieces, air-dried under shade and ground into powder using an electric grinder. A mass of 375 g of powder was exhaustively extracted with 1 l of methanol. After filtration, the solvent was evaporated under reduced pressure in a rotary evaporator at 45°C to afford the methanol extract (47.50 g). An amount of 32.50 g of this extract was pre-dissolved in 100 ml of a mixture of methanol and water (1:9) and then 400 ml of *n*-hexane was added and shaken vigorously. After about 30 min, the *n*-hexane phase was collected and the process repeated thrice. Methanol was then evaporated from the polar phase and the aqueous residue treated sequentially with ethyl acetate and *n*-butanol. The *n*-hexane, ethyl acetate and *n*-butanol were evaporated under reduced pressure in rotary evaporator to afford 4.64, 15.78 and 2.63 g of fractions respectively. The aqueous residue (9.44 g) was obtained after drying the residual portion in the oven at 40°C for 48 h. The methanol extract and fractions were subjected to phytochemical screening using standard procedures [12].

### Fractionation and isolation

A quantity of 10.5 g of the ethyl acetate fraction was subjected to silica gel 60 (0.20-0.500 mm) flash chromatography and eluted with mixtures of *n*-hexane (Hex) and ethyl acetate (EtOAc) of increasing polarity (0-100%) to yield a total of 9 fractions of 200 ml each. These fractions were combined on the basis of TLC profiles into four major fractions: F1 [4.77 g, Hex/EtOAc (100:0), (80:20), (70:30)], F2 [2.48 g, Hex/EtOAc (60:40), (50:50)], F3 [1.56 g, Hex/EtOAc (40:60)] and F4 [0.63 g, Hex/EtOAc (30:70), (20:80), (0:100)]. Fraction F1 was further dissolved in a mixture of Hex and EtOAc (60:40) and a whitish compound precipitated. It was filtered and rinsed with EtOAc to afford compound **1 **(100 mg). The filtrate (4.5 g) was subjected to further silica gel column chromatography (0.063-0.200 mm) to afford compound **2 **(113 mg). Fraction F3 afforded compound **3 **(108 mg) as a yellowish powder from the mixture Hex/EtOAc (60:40). Fraction F4 was further subjected to column chromatography (0.063-0.200 mm) purification using Hex and EtOAc (40:60) to afford compound **4 **(54 mg).

### Chemical analysis

The *n*-hexane fraction was subjected to GC-MS using an Agilent 6890N Network GC system/5975 Inert × L Mass selective Detector at 70 eV and 20°C. The GC column was a CP-S-il 8 CB LB, fused silica capillary column (0.25 mm × 30 m, film thickness 0.25 μm). Helium was used as carrier gas at a flow rate of 1.2 ml/min. The injector port was maintained at 250°C; the oven temperature was programmed at 5°C/min from 70°C to 300°C. A solution of each fraction was prepared in chloroform at a concentration of 10% (wt/v). To 90 μl of this solution, 10 μl of trimethyl sulfonium hydroxide (TMSH) was added and 1 μl of the resulting mixture was injected into the GC -MS apparatus. The constituents were identified by comparing their mass spectra data with those stored in NIST05 and Wiley237 database libraries.

Aluminium sheet pre-coated with silica gel 60 GF_254 _(Merck) was used for thin layer chromatography (TLC). The spots were visualized under UV light (254 and 366 nm) with a UV lamp model 52-58 mineralight, and sprayed with 50% aqueous solution of H_2_SO_4 _followed by heating at 100°C.

IR spectra were measured with KBr disks using FT-IR-8400 S Shimadzu spectrophotometer. EI-MS were carried out on a GCT Premier CAB109 TOF mass spectrometer. ^1^H-, ^13^C-NMR and 2D-NMR (COSY ^1^H-^1^H, HMBC and HSQC) spectra were recorded in acetone-*d_6 _*(500 MHz for ^1^H and 125 MHz for ^13^C) on a Brücker-Avance-500 MHz NMR spectrometer.

(5*S*,6*R*,8a*R*)-5-(carboxymethyl)-3,4,4a,5,6,7,8,8a-octahydro-5,6,8a-trimethylnaphthalenecarboxylic acid (**1**): White powder; ^13^C NMR (100 MHz, CDCl_3 _+ CD_3_OD): *δ *14.9 (5-Me), 17.8 (C-4), 20.7 (6-Me), 21.0 (8a-Me), 25.3 (C-7), 27.0 (C-3), 29.4 (C-8), 35.2 (C-6), 37.5 (C-8a), 38.5 (C-5), 43.6 (C-1'), 44.6 (C-4a), 136.9 (C-2), 142.6 (C-1), 169.3 (1-COOH), 175.4 (C-2'); FABMS: *m/z *279 ([M - H]^+^, 98), 153 (100), 151 (44), 46 (13); HRFABMS: *m/z *279.1592 (calcd. for C_16_H_23_O_4_: 279.1596).

Methyl 3,4,5-trihydroxybenzoate (**2**): Colorless needles; ^13^C NMR (100 MHz, CDCl_3_): *δ *51.7 (OCH_3_), 109,8 (C-2/C-6), 125.2 (C-1), 148.4 (C-3/C-5), 138.9 (C-4), EIMS: *m/z *184 ([M]^+^, 99), 153 (100), 125 (14), 107 (2), 79 (3); HREIMS: *m/z *184.0370 (calcd. for C_8_H_8_O_5_: 184.0372).

Benzene-1,2,3-triol (**3**): White powder; ^13^C NMR (100 MHz, CDCl_3_): *δ *110.2 (C-4/C-6), 124.6 (C-5), 138.5 (C-2), 148.8 (C-1/C-3); EIMS: *m/z *126 ([M]^+^, 100), 108 (21), 80 (24), 52 (27); HREIMS: *m/z *126.0312 (calcd. for C_6_H_6_O_3_: 126.0317).

2,3-dihydroxypropyltriacontanoate (**4**): Whitish gum; ^13^C NMR (125 MHz, CDCl_3_): *δ *14.2 (CH_3_), 22.7-34.2 (28 CH_2_), 63.4 (C-3), 65.2 (C-1), 70.3 (C-2), 172.3 (COO); EIMS: *m/z *43 (100), 57 (80), 71 (44), 83 (64), 85 (28), 97 (69), 99 (7), 111 (37), 125 (19), 127 (3), 139 (8), 141 (3), 153 (5).

### Microorganisms and growth conditions

The microorganisms used in this study consisted of two Gram (+) bacteria (*Enterococcus faecalis *ATCC 10541 and *Staphylococcus aureus *ATCC 25922); six Gram (-) bacteria *(Pseudomonas aeruginosa *ATCC 27853, *Escherichia coli *ATCC 11775, *Klebsiella pneumoniae *ATCC13883, *Salmonella typhi *ATCC 6539, *Proteus mirabilis *and *Shigella flexneri)*; and 10 yeasts (*Candida albicans *ATCC 9002, *C. albicans *ATCC 2091, *C. albicans ATCC 24433, C. parapsilosis *ATCC 22019, *C. lusitaniae *ATCC 200950, *C. tropicalis *ATCC 750, *C. krusei *ATCC 6258, *C. guillermondi, C. glabbrata *IP 35 and *Cryptococcus neoformans *IP 95026). The reference strains (ATCC) were obtained from American Type Culture Collection (Rockville, USA). The two clinical bacterial isolates were collected from "Centre Pasteur" (Yaoundé, Cameroon) and the two IP fungal strains were obtained from "Institute Pasteur" (Paris, France). The bacterial and fungal strains were grown at 35°C and maintained on nutrient agar (NA, Conda, Madrid, Spain) and Sabouraud Dextrose Agar (SDA, Conda) respectively.

### Antimicrobial assays

The minimum inhibitory concentration (MIC) of the crude methanol extract, fractions and isolated compounds were determined through broth microdilution method in 96-well micro-titre plates as described by Zgoda and Porter [13]. The 96-well plates were prepared by dispensing into each well 100 μl of Mueller Hinton broth for bacteria and Sabouraud Dextrose broth for yeasts. The test substances were initially prepared in 10% ethanol/tween 80 in broth medium at 3124.8 μg/ml (methanol extract and fractions), 1250 μg/ml (isolated compounds) and 50 μg/ml (reference antibiotics). A volume of 100 μl of each test sample was added into the first wells of the micro-titre plate. Serial two-fold dilutions of these test samples were made and 100 μl of inoculum standardized at 10^6 ^CFU/ml for bacteria or 2.5 × 10^5 ^CFU/ml for yeasts (at 600 nm, Jenway 6105 UV/Vis spectrophotometer- 50 Hz/60 Hz) [14] was then added into each well. The last wells (N°12) served as sterility controls (contained broth only) or negative control (broth plus inoculum). This gave final concentration ranges of 781.25-0.76 μg/ml, 312.50-0.30 μg/ml and 12.50-0.01 μg/ml for the methanol extract or fractions, isolated compounds and reference substances respectively. The plates were sealed with parafilm, then agitated with a plate shaker to mix their contents and incubated at 35°C for 24 h for bacteria and 48 h for yeast.

The MICs of each test sample was detected following addition of 50 μl (0.2 mg/ml) *p*-iodonitrotetrazolium chloride (INT, Sigma-Aldrich, South Africa) solution for bacteria. Viable bacteria reduced the yellow dye to a pink colour. For yeast, MICs were determined by visualising the turbidity of the wells. The MIC corresponded to the lowest well concentration where no colour or turbidity change was observed, indicating no growth of microorganism. The MBC or MFC was determined by adding 50 μl aliquots of the clear wells to 150 μl of freshly prepared broth medium and incubating at 35 ^°^C for 48 h. The MBC or MFC was regarded as the lowest concentration of test sample which did not produce a colour or turbidity change as above. All tests were performed in triplicates.

### DPPH radical scavenging activity

Radical scavenging activity of test samples (methanol extract, fractions and isolated compounds) was determined spectrophotometrically (Jenway, spectrophotometer model 1605) at 517 nm under UV/Visible light using DPPH radical [15]. The methanol extract and fractions, isolated compounds, and L-ascorbic acid were prepared in methanol and tested at concentration ranges of 200 to 6.25 μg/ml, 64 to 0.25 μg/ml and 8 to 0.25 μg/ml respectively. A volume of 900 μl of DPPH^· ^solution (20 mg/l) was mixed with 100 μl of test sample in a curve and the absorbance (Ab) was read immediately and after 30 min incubation at room temperature (As). The experiments were carried out in triplicate. The percentages of DPPH^· ^scavenged (RSa %) by test samples were calculated as:

The radical scavenging activity fifty (RSa_50_) corresponding to the amount of sample necessary to decrease by 50% the amount of free radical DPPH was determined by plotting the scavenging activity against the logarithm of sample concentration [16].

### Statistical analysis

The data on antioxidant activity were subjected to the one-way analysis of variance (ANOVA) and results were expressed (where appropriate) as mean ± standard deviation. Differences between means of samples were compared using Duncan's multiple range tests at *P <*0.05.

## Results and discussion

### Chemical composition and antimicrobial activity

**Figure 1:**
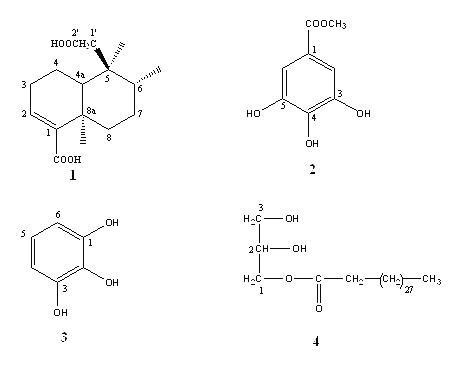
**Chemical structures of compounds isolated from *Entada abyssinica *ethyl acetate fraction**. **1**:(5*S*,6*R*,8a*R*)-5-(carboxymethyl)-3,4,4a,5,6,7,8,8a-octahydro-5,6,8a-Trimethylnaphthalenecarboxylic acid; **2**: methyl 3,4,5-trihydroxybenzoate; **3**: benzene-1,2,3-triol; **4**: 2,3-dihydroxypropyltriacontanoate.

The qualitative analysis of the methanol extract and fractions of *E. abyssinica *revealed the presence of alkaloids, flavonoids, tannins, saponins and cardiac glycosides (Table 1). These results show a similarity in chemical composition between *Entada abyssinica *and *Entada africana *as well as *Entada phaseoloides *[17]. Lipid components identified in *n*-hexane fraction were mainly saturated and unsaturated fatty acids (Table 2). The structures of the isolated compounds from *E. abyssinica *were established by spectroscopic analysis [IR, EI-MS, H^1 ^and C^13 ^NMR spectra in conjunction with 2D experiments (COSY ^1^H-^1^H, HMBC and HSQC)] and direct comparison with published data. The compounds were identified as: (5*S*,6*R*,8a*R*)-5-(carboxymethyl)-3,4,4a,5,6,7,8,8a-octahydro-5,6,8a-trimethylnaphthalenecarboxylic acid (**1**) [18]; methyl 3,4,5-trihydroxybenzoate (methyl gallate) (**2**) [19]; benzene-1,2,3-triol (pyrogallol) (**3**) [20] and 2,3-dihydroxypropyltriacontanoate (**4**) (Figure 1).

**Table 1:** Phytochemical screening of *E. abyssinica *methanol extract and fractions

Groups of chemical constituents	Methanol extract	*n*-hexane fraction	Ethyl acetate fraction	*n*-butanol fraction	Aqueous residue fraction
Alkaloids	**+**	**+**	**+**	**+**	**+**
Flavonoids	**+**	**+**	**+**	**+**	**+**
Coumarins	**+**	**-**	**+**	**+**	**+**
Phenols	**+**	**-**	**+**	**+**	**+**
Tannins	**+**	**-**	**+**	**+**	**+**
Saponins	**+**	**-**	**+**	**+**	**+**
Steroids	**-**	**-**	**-**	**-**	**-**
Chalcones	**+**	**-**	**+**	**+**	**+**
Terpernoids	**+**	**-**	**+**	**-**	**-**
Cardiac glycosides	**+**	**-**	**-**	**+**	**+**

+: presence; **-**: absence.

**Table 2:** Percentage composition of chemical constituents in *n*-hexane fraction of *E.abyssinica *stem bark

Chemical constituents/Molecular ion (*m/z*)	Chemical structure	%
Hexadecanoic acid (C_16 _H_32 _O_2_)	CH_3_-CH_2_-[CH_2_]_12_-CH_2_-COOH	17.86
9,12-Octadecadienoic acid (*Z,Z*) (C_18 _H_32 _O_2_)	CH_3_-[CH_2_]_4_-CH = CH-CH_2_-CH = CH-[CH_2_]_7_-COOH	21.14
9-Octadecenoic acid (*Z*) (C_18 _H_34 _O_2_)	CH_3_-[CH_2_]_6_-CH_2_-CH = CH-[CH_2_]_7_-COOH	27.78
Octadecanoic acid (C_18 _H_36 _O_2_)	CH_3_-CH_2_-[CH_2_]_14_-CH_2_-COOH	2.92
298(M+ 1), 257(5), 189(26), 121(30), 107(60), 95(100), 81(54), 55(71), 41(46), 29(10)	nd	4.27
327(M+ 1), 290(2), 274(7), 189(31), 175(16), 120(63), 107(74), 95(100), 55(48), 41(46)	nd	8.50
		
347(M+ 1), 330(77), 315(30), 256(15), 203(36), 175(49), 139(100), 119(57), 107(75), 95(82), 79(64), 55(68), 41(55)	nd	17.50
Total		99.97

nd: not determined.

The results of the antibacterial and antifungal activities of the methanol extract, fractions and isolated compounds are reported in Table 3. These substances displayed varied antibacterial and antifungal activities across the studied pathogens. The observed wide range of antimicrobial properties for the methanol extract and fractions can be explained by the presence of various groups of potentially active classes of secondary metabolites (Table 1). Indeed flavonoids [21], saponins [22], polyphenols [23], cardiac glycosides [24], coumarines [25], tannins [26], triterpenes and alkaloids [27,28] have been reported to possess antimicrobial activities. It was noted that the bacterial agents were more susceptible to the tested samples than yeasts. Yeasts like other fungi are eukaryotic organism with more complex structural organisation compared to the simple prokaryotic bacterial cells. This probably explains the difference in sensitivity of these two groups of microorganisms. According to the classification of Rios and Rico [29], the methanol extract and fractions (MIC range of 24.4 to 781.2 μg/ml) could be considered to possess significant (MIC < 100 μg/ml), moderate (100 < MIC = 625 μg/ml) or weak (MIC > 625 μg/ml) activity against the corresponding pathogens. The antibacterial activity of the methanol extract of *E. abyssinica *stem bark reported in this study are generally high compared to that of Fabry et al. [30]. The *n*-hexane fraction acted most on bacteria. Its major component, octadecenoic acid, has been reported to possess antibacterial and antifungal activities [31]. Moreover, this does not exclude the fact that the observed antibacterial activity could be a result of the combined effect of all the detected lipid components in the *n*-hexane fraction [32]. The antibacterial mechanism of action of fatty acids is still poorly understood, however, the prime target of these compounds is the cell membrane, where they interfere with the electron transport chain and oxidative phosphorylation [33]. The ethyl acetate and *n*-butanol fractions were generally more effective on both bacteria and yeasts, while the aqueous residue fraction displayed greater activity on yeasts.

**Table 3:** Minimal inhibitory concentration (MIC)/minimum bactericidal or fungicidal concentration (MBC or MFC) of test substances (μg/ml)

Microorganisms	Parameters	Test samples^a^									
		ME	HF	EF	*n*B	AR	1	2	3	4	Ref
**Gram (-) bacteria**											
*Pseudomonas *	MIC	195.3	24.4	781.2	390.6	781.2	39.0	9.7	9.7	312.5	1.5
*aeruginosa*	MBC	781.2	390.6	na^b^	na	na	156.2	19.5	9.7	> 312.5	12.5
*Proteus *	MIC	195.3	390.6	390.6	781.2	na	156.2	19.5	39.0	na	0.3
*mirabilis*	MBC	781.2	na	390.6	na	na	156.2	39.0	156.2	na	1.5
*Shigella flexneri*	MIC	781.2	48.8	390.6	24.4	390.6	78.1	39.0	39.0	na	0.2
	MBC	na	390.6	390.6	195.3	na	156.2	156.2	78.1	na	6.2
*Klebsiella *	MIC	781.2	24.4	97.6	781.2	na	78.1	19.5	78.1	na	0.2
*pneumoniae*	MBC	na	195.3	390.6	na	na	156.2	39.0	156.2	na	3.1
*Salmonella typhi *	MIC	781.2	390.6	97.6	781.2	na	156.2	39.0	78.1	na	0.3
	MBC	na	na	390.6	na	na	312.5	156.2	156.2	na	1.5
*Escherichia coli*	MIC	781.2	24.4	97.6	24.4	na	78.1	39.0	156.2	312.5	0.2
	MBC	na	390.6	390.6	390.6	na	312.5	312.5	156.2	> 312.5	1.5
**Gram (+) bacteria**											
*Enterococcus *	MIC	195.3	390.6	390.6	781.2	na	78.1	39.0	78.1	na	0.2
*faecalis*	MBC	na	781.2	781.2	na	na	312.5	312.5	156.2	na	1.5
*Staphylococcus *	MIC	390.6	195.3	97.6	781.2	390.6	78.1	39.0	78.1	na	1.5
*aureus*	MBC	na	na	390.6	na	na	39.0	156.2	156.2	na	12.0
**Yeast**											
*C. albicans *	MIC	na	na	195.2	390.6	195.3	312.5	312.5	78.1	na	0.01
ATCC 2091	MFC	na	na	195.3	390.6	1953	312.5	312.5	156.2	na	0.08
*C. albicans *	MIC	781.2	781.2	195.3	195.3	195.3	312.5	156.2	156.2	312.2	0.01
ATCC9002	MFC	na	na	781.2	195.3	390.6	312.5	156.2	156.2	> 312.5	0.08
*C. albicans *	MIC	195.3	390.6	195.3	390.6	195.3	156.2	312.5	78.1	na	0.02
*ATCC 24433*	MFC	781.2	na	195.3	na	195.3	312.5	312.5	156.2	na	0.02
*Candida *	MIC	781.2	781.2	195.3	390.6	390.6	312.5	156.2	78.1	na	0.01
*parapsilosis*	MFC	na	390.6	781.2	390.6	781.2	312.5	156.2	156.2	na	0.08
*Candida *	MIC	195.3	na	48.8	390.6	195.3	312.5	312.5	78.1	na	0.04
*tropicalis*	MFC	781.2	na	390.6	781.2	195.3	312.5	312.5	156.2	na	0.08
*Candida krusei*	MIC	na	781.2	781.2	390.6	195.3	312.5	312.5	78.1	na	0.04
	MFC	na	na	na	390.6	195.3	312.5	312.5	156.2	na	0.04
*Candida glabrata *	MIC	na	781.2	195.3	390.6	390.6	78.1	312.5	78.1	na	0.02
	MFC	na	na	195.3	na	781.2	78.1	312.5	156.2	na	0.02
*Candida *	MIC	781.2	390.6	195.3	195.3	195.3	156.2	312.5	78.1	na	0.01
*lusitaniae*	MFC	na	781.2	390.6	195.3	195.3	156.2	312.5	78.1	na	0.08
*Cryptococcus *	MIC	390.6	781.2	97.6	390.6	195.3	195.3	78.1	78.1	na	1.00
*neoformans*	MFC	na	na	781.2	390.6	195.3	312.5	156.2	78.1	na	1.00
*Candida *	MIC	781.2	781.2	195.3	390.6	390.6	312.5	156.2	78.1	na	0.01
*guillermondi*	MFC	na	na	na	na	781.2	312.5	156.2	156.2	na	0.08

^a^ME: methanol extract; HF: *n-*hexane fraction; EF: Ethyl acetate fraction; nB: *n*-butanol fraction; AR: Aqueous residue; **1**: (5*S*,6*R*,8a*R*)-5-(carboxymethyl)-3,4,4a,5,6,7,8,8a-octahydro-5,6,8a-Trimethylnaphthalenecarboxylic acid; **2**: methyl 3,4,5-trihydroxybenzoate; **3**: benzene-1,2,3-triol; **4**: 2,3-dihydroxypropyltriacontanoate and Ref (reference drugs): ciprofloxacin for bacteria and nystatin for yeasts.

^b^na: not active at concentrations upto 781.2 μg/ml.

The isolated compounds were equally more active on bacteria than yeasts. Compounds **2 **and **3 **showed greater activity (MIC = 9.7-312.5 μg/ml) than compounds **1 **(MIC = 39.0-312.5 μg/ml) and **4 **(78.12-312.5 μg/ml). To the best of our knowledge, the antimicrobial and antioxidant activities of compound **1 **are reported herein for the first time. The presence of methyl groups on the carbon rings of this compound could be critical for its activity [34]. Compound **2 **is a known medicinally important substance that was previously isolated from *Entada africana *[35], *Acer ginnala *[36] and *Galla rhois*, and has been reported to possess antimicrobial activities [37] and anti-asthmatic effects [38]. The activities of this compound herein reported corroborates those of Jang-Gi et al. [37] with MICs of 250 and 500 μg/ml on resistant bacterial strains. Compound **3 **(pyrogallol) was as well active on both bacteria (MIC of 9.7 to 78.1 μg/ml) and yeasts (MIC of 78.1 to 156.2 μg/ml), this antimicrobial activity was found to be greater in comparison to that reported by Jin et al. [20]. Pyrogallol contains both phenolic hydroxyl groups and a system of delocalized electrons, conditions critical for its antimicrobial activity [20]. The presence of hydroxyl groups on compounds **2 **and **3 **could be responsible for the observed antimicrobial activity. Compound **4 **recorded weak and selective antimicrobial activities against the tested microorganisms and these activities could be due to the 2,3-dihydroxypropyl moiety [39].

### Antioxidant activity

The free radical scavenging activities of samples (RSa_50_) are reported in Table 4. The ethyl acetate fraction (RSa_50 _of 3.07 μg/ml) showed the greatest activity while the *n*-hexane fraction had the least (RSa_50 _of 35.87 μg/ml). Phenols, flavonoids, saponins and tannins identified in the extract and fractions may be responsible for the observed antioxidant activities [40]. Compounds **2**, **3 **(phenolic compounds) and **4 **(fatty ester) demonstrated interesting radical scavenging activities (RSa_50 _of 1.45, 1.45 and 1.60 μg/ml respectively. The antioxidative effects of phenolic compounds (**2 **and **3**) are mainly due to their redox properties, which can play an important role in absorbing and neutralizing free radicals, quenching singlet and triplet oxygen, or decomposing peroxides [40]. It is known that the antioxidant activity of an aromatic compound is proportional to the number of hydroxyl groups it contains [41]. This probably explains the high radical scavenging activities of compounds **2 **and **3**. The activity of **2 **in this assay was higher than that (RSa_50 _= 2.8 μg/ml) reported by Seong et al. [37] against DPPH free radical.

**Table 4:** Test sample's concentration reducing 50% of free radical DPPH (RSa_50_)

Test samples	RSa_50 _(μg/ml)
***Plant extract and fractions***	
Methanol extract	7.12 ± 0.21*
*n*-hexane fraction	35.87 ± 0.07*
Ethyl acetate fraction	3.07 ± 0.04*
*n*-butanol fraction	3.10 ± 0.10*
Aqueous residue fraction	7.15 ± 0.08*
***Isolated compounds*^a^**	
**1**	298 ± 0.57*
**2**	1.45 ± 0.00*
**3**	1.45 ± 0.00*
**4**	1.60 ± 0.01*
***Reference substance***	
L-ascorbic acid	0.68 ± 0.00

^a^**1:**(5*S*,6*R*,8a*R*)-5-(carboxymethyl)-3,4,4a,5,6,7,8,8a-octahydro-5,6,8a-Trimethylnaphthalenecarboxylic acid; **2**: methyl 3,4,5-trihydroxybenzoate; **3**: benzene-1,2,3-triol and **4**: 2,3-dihydroxypropyltriacontanoate.

*Scavenging activity significantly lower than the reference, Duncan (p < 0.05).

## Conclusion

The results obtained from this study reveal that the stem bark of *E. abyssinica *may be useful in the development of an antimicrobial phytomedicine which can be standardised using the isolated compounds.

## Competing interests

The authors declare that they have no competing interests.

## Authors' contributions

GNT and PKL carried out the assays in this study; HKP participated to extract fractionation and isolation of compounds; HK and YO participated to structural elucidation of compounds **1 **and **2; **GV and GG YO participated to structural elucidation of compounds **3 **and **4**; JRK designed and supervised the work along with the manuscript writing. All authors read and approved the final manuscript.

## Pre-publication history

The pre-publication history for this paper can be accessed here:

http://www.biomedcentral.com/1472-6882/11/57/prepub
